# Supplementary figures and images for: Immunosuppressive drugs and diet interact to modify the gut microbiota and cardiovascular risk factors, and to trigger diabetes
Source: PLoS One. 2025 Mar 28;20(3):e0320438. doi: 10.1371/journal.pone.0320438 (PMC11952260; doi:10.1371/journal.pone.0320438)

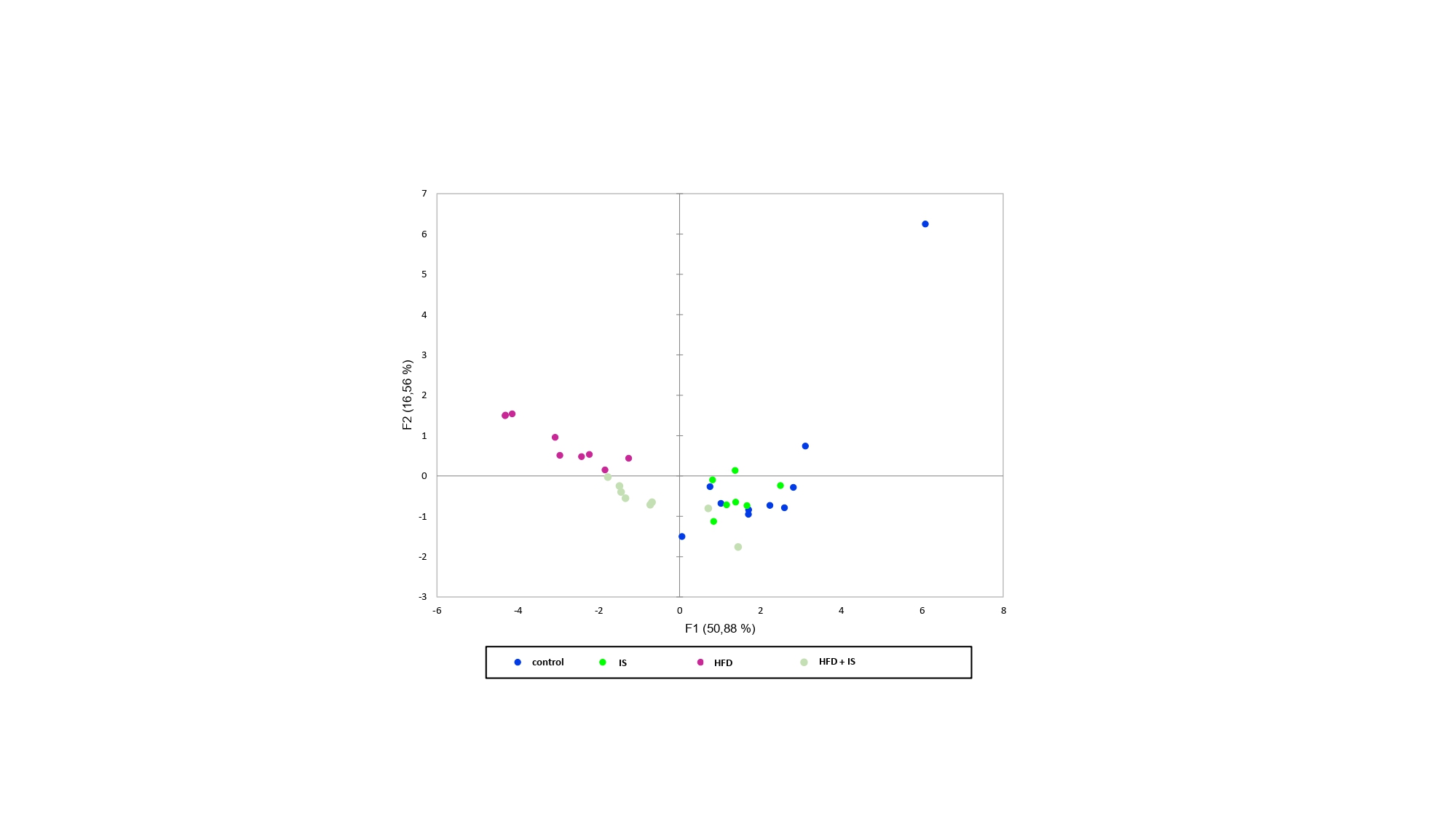

Supplement: S2 Fig — The overall composition of the gut microbiota as assessed by qPCR quantification of specific bacteria was significantly different in the high fat diet group compared to the standard diet group, with or without immunosuppressive drugs (segregation on the F1 axis). However, the adjunction of immunosuppressive drugs to the high fat diet modulated the overall microbiota composition. Indeed, the HFD and HFD+IS groups were not segregated by the F1 axis but by the F2 axis. These results were similar to those obtained with the 16S rDNA sequencing of gut microbiota. (JPG) [file pone.0320438.s002.jpg]

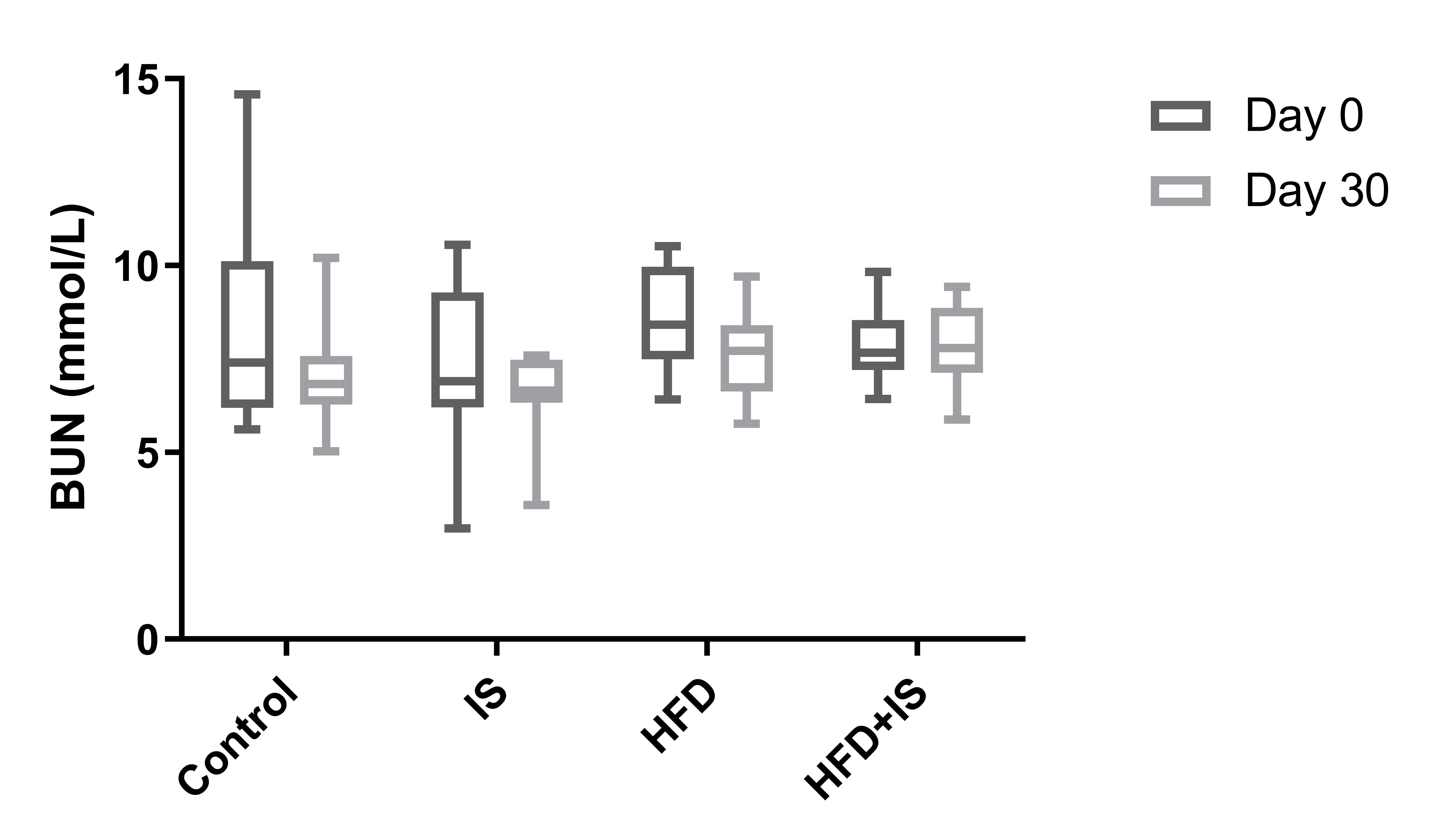

Supplement: S3 Fig — BUN was not significantly different throughout the experiment and between groups. BUN: Blood urea nitrogen. (JPG) [file pone.0320438.s003.jpg]
