# Supplementary material for: Immunosuppressive drugs and diet interact to modify the gut microbiota and cardiovascular risk factors, and to trigger diabetes
Source: PLoS One. 2025 Mar 28;20(3):e0320438. doi: 10.1371/journal.pone.0320438 (PMC11952260; doi:10.1371/journal.pone.0320438)
Supplement: Attachment — Submitted filename: Response to reviewers.docx [file pone.0320438.s004.docx]

Editor

As raised by one reviewer, your observatioins on the effect of HFD is not very clear. You need at least to rerun some measurments on plasma TG and Cholesterol levels.

≫ The lipid values are in international units, mmol/L and not in mg/dL as stated on the graph. We apologize for this mistake but there is nothing wrong with the dosages that we performed. The graph now shows mmol/L.

It seems that your HFD is only for 4 weeks which is quite short in order to increase plasmatic TG and CHOL levels. You have to clearly justify the duration of the diet. In this situation, you cannot speak about combine effects of HFD and IS.

≫ Even though 4 weeks seems a short duration for the diet and the IST, we were able to observed clear-cut differences between groups. We chose this duration because new onset diabetes, weight gain, HBP, dyslipidemia are observed rapidly after KT as a consequence of IST. Also, we wanted to distinguish direct effects of diet and IST from long terms effect that maybe more related to weight gain and insulin resistance. We added a comment in the discussion.

Reviewer #1: General comment

The values for total cholesterol and triglycerides presented in the manuscript are not appropriate for C56Bl6 mice (please see, DOI:10.1016/j.yexcr.2015.07.032) therefore you should re-run all the measurements and the statistical analyses, as well.

≫ The lipid values are in international units, mmol/L and not in mg/dL as stated on the graph. We apologize for this mistake but there is nothing wrong with the dosages that we performed. The graph now shows mmol/L.

Additionally, the data reported about the loss of BW indicate that the drug treatment was toxic for the mice. The Authors have included a sentence in this regards in the Discussion, but I believe that is not sufficient because they did not take into account this possibility.

≫ We have added IS drug blood dosages in the discussion. Based on these dosages, we explain why we don’t think that IST were toxic in our experiments.
