# Supplementary material for: Immunosuppressive drugs and diet interact to modify the gut microbiota and cardiovascular risk factors, and to trigger diabetes
Source: PLoS One. 2025 Mar 28;20(3):e0320438. doi: 10.1371/journal.pone.0320438 (PMC11952260; doi:10.1371/journal.pone.0320438)
Supplement: Attachment — Submitted filename: Rebuttal letter.docx [file pone.0320438.s005.docx]

Rebuttal letter

Reviewer #2:

After analyzing this manuscript, it can be considered for publication.

≫ Thank you very much

Reviewer #3: This study showed that immunosuppressive therapy (IST) and high-fat diet (HFD) had some effects on gut microbiota and metabolism in mice. Here are some comments.
1. It could be improved by mentioning the specific immunosuppressive drugs used in the Abstract.

≫ This precision was added to the revised manuscript

2. Note the units of insulin concentration, which should be consistent in the main text and Figure 1; The ordinate of Figure 5 is consistent with the main text.

≫ We apologize for this mistake and sincerely thank reviewer #3 for noticing it. The unit for insulin concentration is now consistent in the text and the figure: mUI/L.

3. LPS on line 247 is not equivalent to endotoxemia

≫ This was specified in the main text

4. Specify the exact IST regimen used (prednisone, MMF, tacrolimus, etc.) and their doses.

≫ We believe that this is already detailed in the paragraph “diets and treatment administration” of the material and methods section

5. Provide more details on the HFD composition

≫ This was added to the manuscript

6. Clarify the duration of the experiment and the time points for data collection

≫ This has been detailed in the material and methods section of the present revision

7. Include more statistical details in figure legends.

≫ The statistical details are described in the material and methods section and mostly comprises simple comparison tests. Adding them in the legend of each figure depends on the editorial choices of each journal and this was not asked to us in the first round of revisions.

8. A more in-depth discussion of the mechanisms by which IST and HFD may alter the gut microbiota and impact metabolism is necessary.

≫ “The mechanisms by which IST and HFD may alter the gut microbiota and impact metabolism” is a whole field of research *per se* and is way beyond the scope of the research presented here. Consequently, we believe that describing these mechanisms more in details would only be speculative as we did not investigate them, and wouldn’t add any scientific value to our work. However, we have already cited several works that specifically address these questions in the introduction and the discussion (see references 6-8, 13, and 31-34).

9. A discussion of the limitations of the study and potential areas for future research is necessary.

≫ This is the second round of revisions. A discussion of the limits of our work was already included in the original submission. It was extended in the first revision of the manuscript. Please tell us if you believe that a specific limitation has not been addressed in the last paragraph of the discussion.

As for future research, we specify in the manuscript that microbiota transfer experiments are underway. We believe that the goal of a scientific paper is more to describe what has been done than what is intended to be done.
